# Supplementary material for: Factors correlated with visual outcomes at two and four years after vitreous surgery for proliferative diabetic retinopathy
Source: PLoS One. 2021 Jan 14;16(1):e0244281. doi: 10.1371/journal.pone.0244281 (PMC7808600; doi:10.1371/journal.pone.0244281)
Supplement: Attachment — Submitted filename: Reviewer Comments.docx [file pone.0244281.s002.docx]

Dear authors

I would like to congratulate you on your work. The findings and results are important and can help surgeons make the right decisions. The advantages of the study are: large cohort of patients, long follow up and the fact that only two vitreoretinal surgeons performed surgeries.

The study has some major limitations:

- **Effect of gauges on vitreoretinal surgery:** There is considerable talk about functional and morphological results after vitreoretinal surgery performed with the help of 20G, 23 G , 25 G but no analysis on the effect of the different gauges. Did the evolution of vitreoretinal surgery have any effect on the results?
- **No information on treatment regiments before and after surgery:** Patients with PDR will most certainly need treatment with injection and perhaps laser treatment before and after surgery. There is only one short mention on the use of Triamncinolone before surgery but no mention on the use of injections or laser before and after surgery. I believe this is a very important part that will judge the anatomical and functional results and has to be mentioned with sub-analysis in each group. (lower initial BCVA means more or less post OP treatment with injections or laser?)
- **Did the status of the fibrovascular membranes and the status of the posterior hyaloid play a role in the functional and morphological results?** Did the manipulation of these mambranes have an effect on the RD rate and final BCVA?
- **Lines 111 and 112 Explain:** Among them, 91 eyes of 70 patients could be followed up for 4 years after 112 a primary surgery, and 37 eyes of 30 patients could be followed up for 2-3 years How is it possible to have data on 91 eyes with a 4 year follow up and only 37 eyes with a follow up up to 2-3 years. Is it opposite correct? What do you mean with 2-3 years? Not precise enough
- **Lines 153 to 160:** A statistical association between initial BCVA and improvement of vision after 2-3 year and 4 year is essential! Which patients profited more from surgery? Does initial BCVA is a prognostical factor?
- **Discussion Line 214-220:** Important and helpful results but these results are incomplete if the treatment regiments before and after surgeries are not elaborated (did patients with rubeosis iridis receive any or more post OP injections or laser treatments compared to patients with no rubeosis?)
